# The changes of coagulation profiles in Kawasaki disease and its associations with clinical classification, intravenous immunoglobulin responsiveness and coronary artery involvement

**DOI:** 10.1007/s10238-024-01430-z

**Published:** 2024-08-06

**Authors:** Dao Ting Li, Qian Yang, Cai Yun Xia, Yan Fang Zhang, Ying Cai, Shu Qi Wu, Qi Jiang, Peng Hu

**Affiliations:** Department of Paediatrics, The First Affiliated Hospital of Anhui Medical University, No. 218 Ji-Xi Road, Hefei, 230022 Anhui Province People’s Republic of China

**Keywords:** Coronary artery, D-dimer, Intravenous immunoglobulin, Kawasaki disease, Multisystem inflammatory syndrome in children

## Abstract

Coagulation disorders are common in Kawasaki disease (KD). The main objectives of the present study were to probe the associations of coagulation profiles with clinical classification, IVIG responsiveness, coronary artery abnormalities (CAAs) in the acute episode of KD. A total of 313 KD children were recruited and divided into six subgroups, including complete KD (n = 217), incomplete KD (n = 96), IVIG-responsive KD (n = 293), IVIG-nonresponsive KD (n = 20), coronary artery noninvolvement KD (n = 284) and coronary artery involvement KD (n = 29). Blood samples were collected within 24-h pre-IVIG therapy and 48-h post-IVIG therapy. Coagulation profiles, conventional inflammatory mediators and blood cell counts were detected. Echocardiography was performed during the period from 2- to 14-day post-IVIG infusion. In addition, 315 sex- and age-matched healthy children were enrolled as the controls. (1) Before IVIG therapy, coagulation disorders were more prone to appear in KD patients than in healthy controls, and could be overcome by IVIG therapy. FIB and DD significantly increased in the acute phase of KD, whereas reduced to normal levels after IVIG therapy. (2) PT and APTT were significantly longer in patients with complete KD when compared with their incomplete counterparts after IVIG therapy. (3) The larger δDD, δFDP and the smaller δPT, δINR predicted IVIG nonresponsiveness. (4) The higher δDD and δFDP correlated with a higher risk for CAAs (DD: *r* = −0.72, FDP: *r* = −0.54). Coagulation disorders are correlated with complete phenotype, IVIG nonresponsiveness and CAA occurrence in the acute episode of KD, and can be rectified by synergistic effects of IVIG and aspirin.

## Introduction

Kawasaki disease (KD) is an acute febrile syndrome of unknown etiology that predominantly affects younger children. Based on the dataset from Japan’s 25th nationwide KD survey, the incidence of KD is 359 per 100,000 children aged 0–4 years, and 7.6% of cases are combined with coronary artery abnormalities (CAAs) [1]. Although the exact pathogenesis of KD remains unclear, it is generally accepted that an exaggerated immune or inflammatory activation triggered by infectious agents may occur in genetically susceptible children who subsequently develop KD. Lee et al. [2] conducted a genome-wide association study encompassing 622 KD cases, and revealed that B-lymphoid tyrosine kinase and CD40 served as two new susceptibility loci for KD in a Han population residing in Taiwan province, China. In addition, a series of studies from our center have suggested that the increased levels in serum tumor necrosis factor-α (TNF-α), interleukin-6 (IL-6), procalcitonin (PCT) and serum ferritin (SF) are associated with atypical and intravenous immunoglobulin (IVIG)-nonresponsive KD in the acute episode, and even predict a high risk for CAAs thereafter [3–6].

Inflammation triggered by acute infection can lead to activation of coagulation in autoimmune disease. A retrospective study of 105 patients with rheumatoid arthritis (RA) indicated that FIB, DD, and FDP were increased in RA patients and positively correlated with the disease activity of RA [7]. A multinational, multicenter study of 362 patients with systemic juvenile idiopathic arthritis (sJIA)-associated macrophage activation syndrome (MAS) indicated that DD was the sole laboratory biomarker experiencing a percentage change of > 50% between the pre-MAS visit and MAS onset [8]. During the COVID-19 pandemic, serum DD was found to be significantly 1.33-fold higher in multisystem inflammatory syndrome in children (MIS-C) as compared with pediatric non-severe COVID-19 on admission [9]; in addition, a DD value greater than 2144 ng/ml predicted for intensive care unit hospitalization with a sensitivity of 82% and a specificity of 75% [10]. Actually, some features of MIS-C are also seen in KD, particularly in children younger than 5 years old. A website-based study from our center encompassing 50 MIS-C patients from 22 eligible studies showed that 35.7% of them had KD-like features [11]. The purpose of the present study is to describe the associations of coagulation profiles with clinical classification, IVIG responsiveness, CAAs in the acute episode of KD.

## Methods

### Subjects

**Fig. 1:**
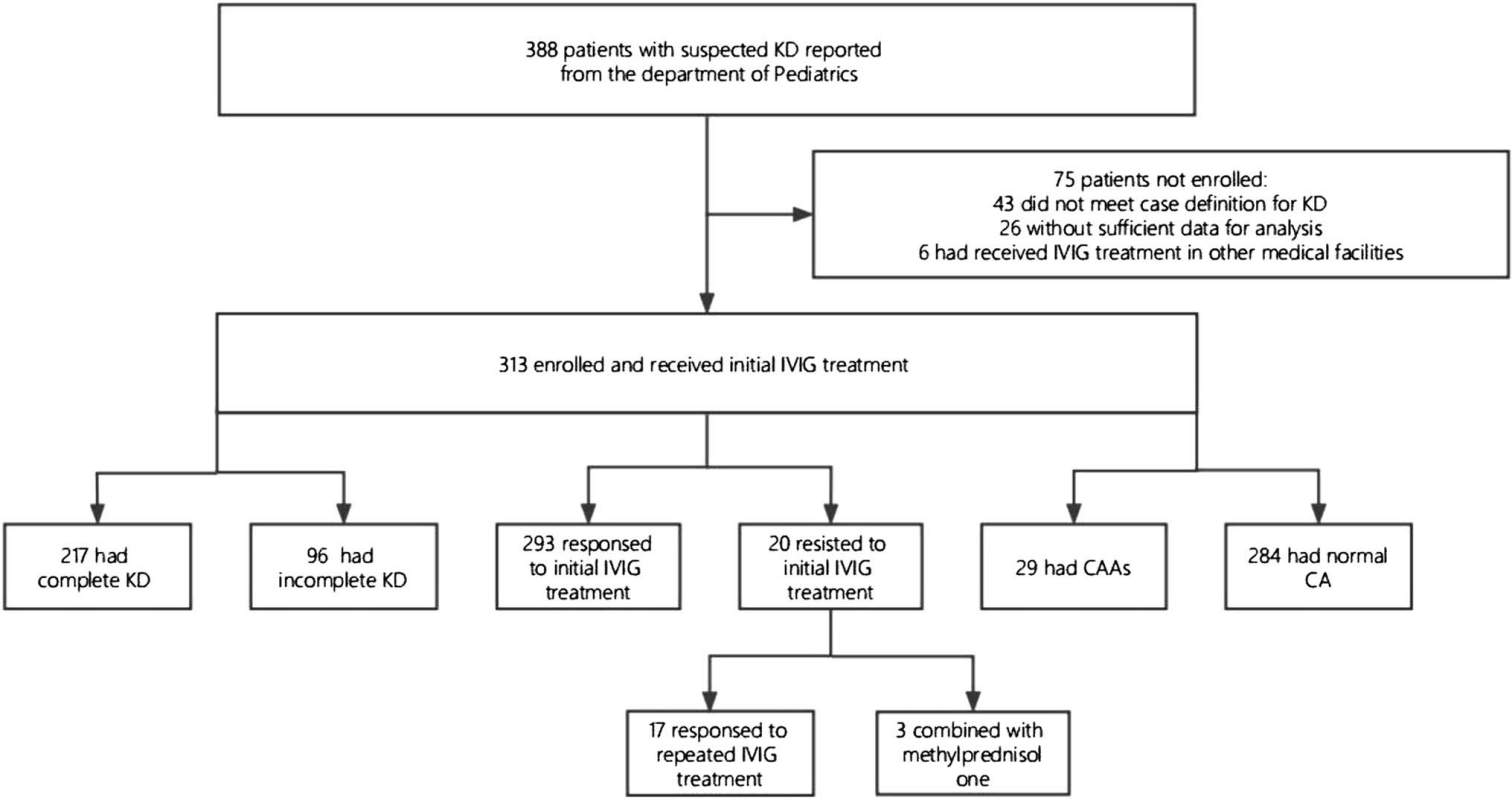
Flowchart of participant selection

The present retrospective study recruited a total of children with KD between July 2015 and December 2022 in Department of Pediatrics, the First Affiliated Hospital of Anhui Medical University. According to the 2017 American Heart Association (AHA) guidelines [12], complete KD is diagnosed in the presence of ≥ 5 days of fever and ≥ 4 of the following five principal clinical features: (1) bilateral conjunctival injection without exudates; (2) changes in the oral mucosa, such as erythema and cracking lips, erythema of the pharynx, and strawberry tongue; (3) changes in extremities, such as redness and swelling in the acute phase, periungual desquamation in the subacute phase; (4) polymorphous exanthema; (5) cervical lymphadenopathy, (≥ 1.5 cm in diameter), usually unilateral. Patients having fever for at least 5 days together with at least 2 of the principal features can be diagnosed as having incomplete KD, if no other disease processes could explain the illness. All patients received IVIG (2 g/kg) and aspirin (30–50 mg/kg/d), within 10 days of the fever onset. Aspirin was given as an anti-platelet agent using 30–50 mg/kg/day at least 48–72 h after cessation of fever. When high-dose aspirin was discontinued, low-dose aspirin (3–5 mg/kg/day) was begun and continued until the patient has no evidence of coronary changes by 6–8 weeks after onset of illness. For children who developed coronary abnormalities, aspirin should be continued indefinitely. Higher-intensity anticoagulation should be considered in those who D-dimer levels elevated to > 5 times the upper limit of normal [13]. IVIG-nonresponsive KD is defined as persistent or recrudescent fever at least 36 h after completion of the first IVIG infusion. One proposed by the Cardiovascular Group of the Chinese Society of Pediatrics classifies coronary arteries as abnormality if the internal lumen diameter is > 3.0 mm in children < 5 years of age or > 4.0 mm in children ≥ 5 years of age, if the internal diameter of a segment measures ≥ 1.5 times that of an adjacent segment, or if the coronary artery lumen is clearly irregular [14] (Fig. 1). In addition, 352 sex- and age-matched healthy children in the same period were enrolled as the controls.

### Laboratory analysis

This study was approved by the Medical Ethic Committee of the First parents. Blood samples were collected from all subjects within 24-h pre-IVIG therapy and 48-h post-IVIG therapy, respectively, including white blood cell counts (WBC), absolute neutrophil counts (ANC), monocytes (MO), C-reactive protein (CRP), erythrocyte sedimentation rate (ESR), red blood cell counts (RBC), hemoglobin (Hb), hematocrit (HCT), platelet (PLT), procalcitonin (PCT), albumin (ALB), gamma-glutamyl transpeptidase (GGT), alanine aminotransferase (ALT), aspartate aminotransferase (AST), total bilirubin (TBil), direct bilirubin (DBIL), indirect bilirubin (IBIL), D-dimer (DD), prothrombin time (PT), international normalized ratio (INR), activated partial thromboplastin time (APTT), fibrinogen (FIB), thrombin time (TT), and fibrin degradation product (FDP).

### Statistical analysis

Normally distributed continuous data were expressed as mean ± standard or median with interquartile range (IQR). Comparisons of the frequencies between groups were analyzed using Chi-square tests, and comparisons among groups were made using ANOVA or Kruskal–Wallis test. Serum data were analyzed using the two independent samples *t* test or Mann–Whitney *U* test, and the paired* t* test or Wilcoxon test for comparison of pre-IVIG to post-IVIG data. Pearson correlation coefficients were reported for DD and other variables of interest. Cutoff value, sensitivity and specificity of DD were identified by receiver operating characteristic (ROC) curve. A value of *P* < 0.05 was considered significant. Statistical analysis was performed using the statistical package for social studies SPSS version 27.0.

## Results

### Demographic features

A total of 313 KD patients (177 males and 136 females) were recruited in this study, with a median (IQR) age of 24.00 [12.00–48.00] months and a range from 2 months to 13 years. In the same period, 315 healthy children (179 males and 136 females) were recruited, with a median (IQR) age of 24.00 [12.00–42.00] months and a range from 2 months to 13 years. Among the KD group, 49 were younger than 1 year of age, including 23 males and 26 females with a median (IQR) age of 6.00 [4.00–9.00] months; 211 were aged 1–5 years, including 121 males and 90 females with a median (IQR) age of 24.00 [12.00–36.00] months; 53 were older than 5 years, including 33 males and 20 females with a median (IQR) age of 84.00 [64.00–96.00] months. In the healthy group, 67 were younger than 1 year of age, including 37 males and 30 females with a mean age of 7.00 [3.00–10.00] months; 192 were aged 1–5 years, including 107 males and 85 females with a mean age of 25.50 [14.00–36.00] months; 56 were older than 5 years, including 35 males and 21 females with a median (IQR) age of 78.50 [67.25–91.75] months. No significant differences were observed in age (*x*^*2*^ = 3.77, *P* > 0.05) and gender (*x*^2^ = 7.00, *P* > 0.05) between KD children and the healthy controls.

### Clinical features

Among 313 KD patients, all five classic diagnostic criteria for KD were met in 93 cases (29.71%), four criteria in 124 (39.62%), three criteria in 60 (19.17%), and two criteria in 26 (8.31%). Therefore, 217 patients (69.33%) had complete KD and 96 patients (30.67%) had incomplete KD. The prevalence rates of clinical diagnostic signs of conjunctivitis, mucous membrane involvement, skin rashes, hands and feet erythema/edema, and cervical lymphadenopathy were 84.30%, 85.60%, 77.30%, 65.20%, and 71.90%, respectively. All patients received IVIG (2 g/kg) within 10 days since the onset of fever and the mean duration of fever before the initial IVIG administration was 6.98 ± 2.38 days. According to the fever duration after the initial IVIG infusion, 293 patients (93.61%) were diagnosed as IVIG responders and 20 patients (6.39%) were identified as IVIG-nonresponders.

All of IVIG-nonresponders received the second dose of IVIG and 3 of them were combined with methylprednisolone simultaneously. Based on the internal diameter of CA, 29 KD patients (9.27%) were defined as having CAAs (left coronary artery: 2.91 ± 0.59 mm; right coronary artery: 3.13 ± 1.11 mm), and 284 patients (90.73%) had normal CA (left coronary artery: 2.01 ± 0.34 mm, right coronary artery: 1.93 ± 0.30 mm). The internal diameters of both coronary arteries were significantly larger in KD patients with CAAs (left coronary artery: *t* = 12.42, *P* < 0.05; right coronary artery:* t* = 13.73, *P* < 0.05), compared with those without CAAs.

### Laboratory findings

Comparisons of laboratory characteristics between KD patients and the healthy controls are shown in Table 1. As for KD patients, WBC, ANC, MO, CRP, ESR, PCT, TBIL, DBIL, IBIL, PT, INR, APTT, FIB, DD and FDP showed a significant decrease, whereas no significant difference was found in RBC, HB, HCT, ALB and AST after IVIG therapy (*P* > 0.05). Before IVIG therapy, KD patients had a significant increase in WBC, ANC, MO, PLT, CRP, ESR, PCT, TBIL, DBIL, ALT, GGT, PT, INR, APTT, FIB, DD and FDP compared with the healthy controls (*P* < 0.05). After IVIG therapy, ANC, FIB and DD in KD patients reduced to normal levels (*P* < 0.05), whereas PLT, ALT, AST, GGT and TT in KD patients still maintained an elevated level and significantly higher than that in the healthy controls (*P* < 0.05).

**Table 1:** Comparison of laboratory characteristics in KD patients and the healthy control groups

Variables	KD patients (n = 313)		Healthy controls (n = 315)	*P* value
	Pre-IVIG	Post-IVIG		
*Age, mo*				
Median (IQR)	24.00 [12.00–48.00]		24.00 [12.00–42.00]	
< 12	6.00 [4.00–9.00]		7.00 [3.00–10.00]	
12–59	24.00 [12.00–36.00]		25.50 [14.00–36.00]	
≥ 60	84.00 [64.00–96.00]		78.50 [67.25–91.75]	
Sex (male/female)	177/136		179/136	
*Laboratory features*				
WBC (10^9^/L) ^*†△#^	13.19 (10.35–16.26)	8.95 (7.57–11.58)	8.26 (6.65–10.01)	^*^ † ^△ #^ *P* < 0.001
ANC (10^9^/L) ^*†#^	8.61 (5.88–11.16)	2.77 (1.71–4.27)	2.66 (1.95–3.58)	^*^ † ^#^ *P* < 0.001
MO^*†△#^	0.71 (0.46–1.00)	0.60 (0.44–0.86)	0.49 (0.37–0.63)	^*^ † ^△ #^ *P* < 0.001
RBC (10^12^/L) ^*†△^	4.22 (3.98–4.47)	4.21 (3.95–4.46)	4.53 (4.25–4.78)	^*^ † ^△^ *P* < 0.001
HB (g/L) ^*†△^	113.00 (106.00–119.00)	112.00 (105.00–119.00)	121.00 (114.00–127.50)	^*^ † ^△^ *P* < 0.001
HCT ^*†△^	33.64 ± 3.19	33.72 ± 3.15	35.81 ± 3.55	^*^ † ^△^ *P* < 0.001
PLT (10^9^/L) ^*†△#^	348.50 (282.00–434.50)	496.00 (409.00–623.00)	316.00 (258.50–371.50)	^*^ † ^△ #^ *P* < 0.001
CRP (mg/L) ^*†△#^	55.06 (24.75–86.75)	5.00 (2.10–9.50)	0.50 (0.50–2.02)	^*^ † ^△ #^ *P* < 0.001
ESR (mm/h) ^*†△#^	61.04 ± 22.89	60.76 ± 21.63	8.92 ± 6.69	^#^*P* = 0.030; ^*^ † ^△^ *P* < 0.001
PCT (ng/ml) ^*†#^	0.33 (0.13–0.82)	0.09 (0.05–0.24)	0.06 (0.05–0.14)	^*^ † ^#^ *P* < 0.001
ALB (g/L) ^*†△^	37.90 (34.90–40.75)	36.30 (33.10–39.10)	44.70 (42.08–47.30)	^*^ † ^△^ *P* < 0.001
TBIL (μmol/L) ^*†△#^	7.15 (5.40–10.08)	5.36 (3.90–7.58)	6.50 (4.86–9.10)	†*P* = 0.024; ^* △ #^ *P* < 0.001
DBIL (μmol/L) ^*†#^	2.30 (1.60–3.58)	1.70 (0.90–3.20)	1.80 (1.19–2.90)	^*^P = 0.005; †*P* = 0.010; ^#^*P* < 0.001
IBIL (μmol/L) ^*†△#^	4.40 (3.21–5.90)	3.30 (2.50–4.40)	5.20 (3.65–6.70)	†*P* = 0.014; ^* △ #^ *P* < 0.001
ALT (U/L) ^*†△#^	29.00 (17.00–59.25)	31.00 (21.00–51.75)	19.00 (14.00–27.00)	^*^ † ^△ #^ *P* < 0.001
AST (U/L) ^*†△^	28.00 (22.00–41.00)	36.50 (31.00–47.00)	34.00 (29.00–43.00)	^△^*P* = 0.018; ^*^ † *P* < 0.001
GGT (U/L) ^*†△#^	23.00 (13.00–70.00)	44.00 (23.50–87.00)	10.00 (8.00–14.00)	^*^ † ^△ #^ *P* < 0.001
PT (s) ^*†△#^	13.70 (12.30–14.80)	11.70 (10.63–12.40)	12.40 (11.00–13.40)	^△^*P* = 0.002; ^*^ † ^#^ *P* < 0.001
PT% ^*†#^	83.10 (75.00–95.00)	103.00 (87.90–120.40)	103.00 (91.60–114.00)	^*^ † ^#^ *P* < 0.001
INR ^*†#^	1.08 (0.99–1.18)	0.95 (0.88–1.01)	0.96 (0.89–1.03)	^*^ † ^#^ *P* < 0.001
APTT (s) ^*†△#^	38.30 (30.40–41.90)	28.35 (25.30–33.20)	35.60 (28.80–40.20)	†*P* = 0.014; ^* △ #^ *P* < 0.001
FIB (g/L) ^*†△#^	5.93 (4.88–6.96)	3.72 (2.89–4.26)	2.52 (2.12–2.98)	^*^ † ^△ #^ *P* < 0.001
TT (s) ^*†△#^	15.90 (15.40–16.70)	18.80 (17.70–19.60)	17.40 (16.30–18.50)	^*^ † ^△ #^ *P* < 0.001
DD (ng/ml) ^*†△#^	1140 (770–1750)	850 (570–1590)	290 (200–410)	^*^ † ^△ #^ *P* < 0.001
FDP (μg/mL) ^*†△#^	4.19 (2.83–6.99)	2.90 (2.04–4.97)	1.20 (0.80–1.74)	^#^*P* = 0.002; ^*^ † ^△^ *P* < 0.001

The measurement data are expressed as mean + standard deviation or median (P25-P75). Abbreviations: WBC, white blood cell counts; ANC, absolute neutrophil counts; MO, monocytes; RBC, red blood cell counts; Hb, hemoglobin; HCT, hematocrit; PLT, platelet; CRP, C-reactive protein; ESR, erythrocyte sedimentation rate; PCT, procalcitonin; ALB, albumin; TBIL, total bilirubin; DBIL, direct bilirubin; IBIL, indirect bilirubin; ALT, alanine aminotransferase; AST, aspartate aminotransferase; GGT, gamma-glutamyl transpeptidase; PT, prothrombin time; INR, international normalized ratio; APTT, activated partial thromboplastin time; FIB, fibrinogen; TT, thrombin time; DD, D-dimer; FDP, fibrin degradation products.^*^P < 0.05, significantly different among three groups; †*P* < 0.05, significantly different between pre-IVIG therapy and the healthy controls; ^△^*P* < 0.05, significantly different between post-IVIG therapy and the healthy controls; ^#^*P* < 0.05, significantly different between pre-IVIG therapy and post-IVIG therapy

### Coagulation profiles and clinical classification

**Fig. 2:**
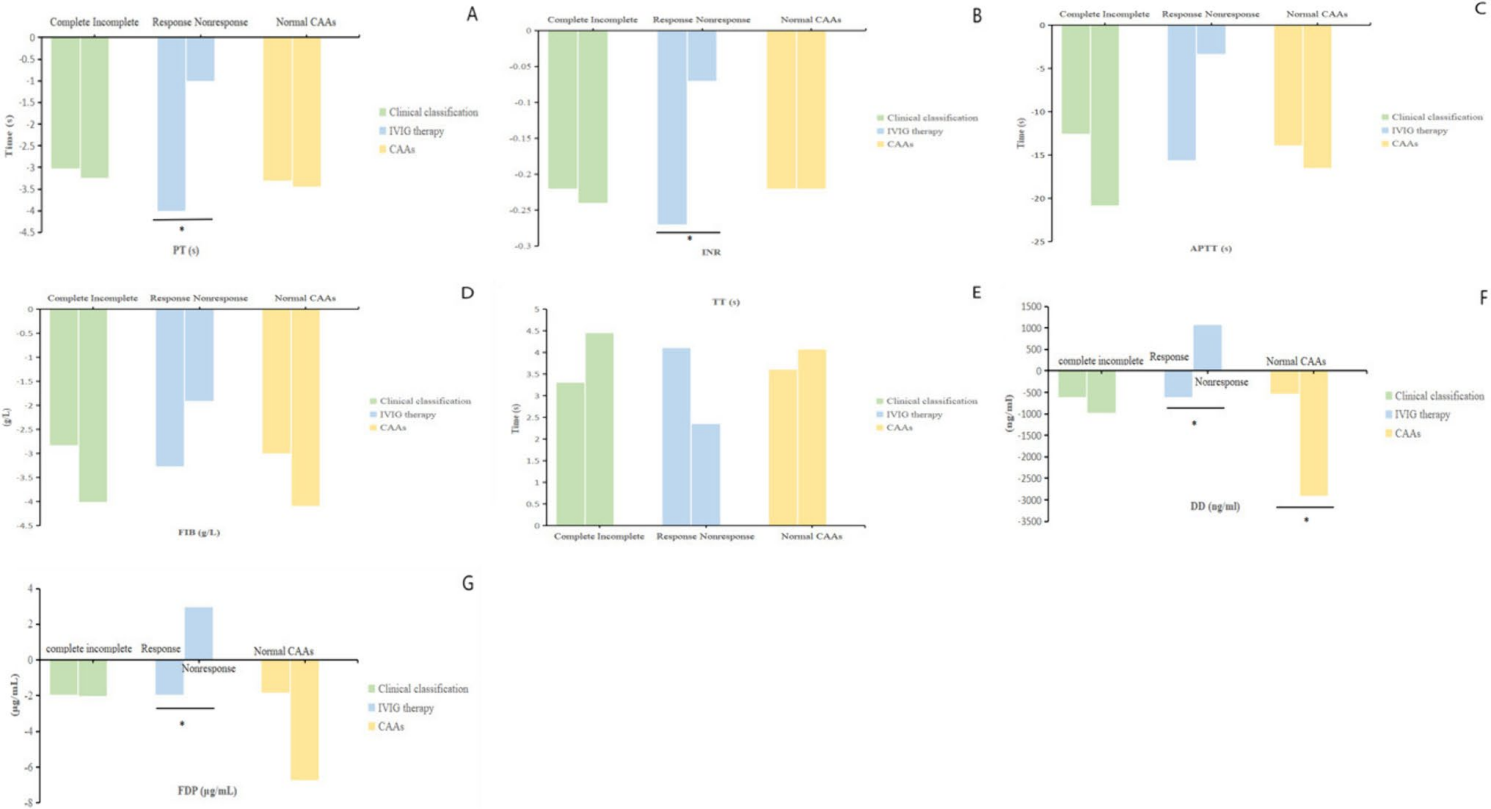
Coagulation profiles in patients with KD stratified by clinical classification, IVIG responsiveness, and coronary artery abnormalities. δPT and δINR were larger in the IVIG-responsive group (Figs. **A** and **B**), and δDD and δFDP were smaller in the IVIG-responsive group when compared with the IVIG-nonresponsive group (Figs. **F** and **G**); the CAA group showed higher δDD than the non-CAA group (Fig. **F**). *P < 0.05, significantly different in delta changes

Comparisons of laboratory characteristics between complete KD patients and incomplete KD patients are shown in Table 2. No significant differences were observed in all laboratory indicators between the two groups before IVIG therapy (*P* > 0.05); PT and APTT were significantly longer in patients with complete KD when compared with their incomplete counterparts after IVIG therapy (*P* < 0.05). Furthermore, to investigate the impact of IVIG on the changes in laboratory indicators between two groups, delta (δ) were defined as the post-IVIG minus the pre-IVIG. The results showed no significant differences in δ of all laboratory indicators between the two groups (*P* > 0.05) (Fig. 2).

**Table 2:** Comparison of laboratory characteristics in patients with different types of KD

Variables	Clinical classification						*P* value
	Complete KD			Incomplete KD			
	Pre-IVIG	Post-IVIG	Delta	Pre-IVIG	Post-IVIG	Delta	
WBC (10^9^/L)	13.07 (10.45,16.00)	8.95 (7.52,11.53)	−3.81 (−6.60,−1.15)	13.45 (10.32,17.19)	9.00 (7.63,11.71)	−3.10 (−7.80,−1.34)	
ANC (10^9^/L)	8.68 (5.99,11.18)	2.61 (1.67,4.20)	−5.86 (−7.95,−2.85)	8.60 (5.56,11.11)	3.15 (1.76,4.44)	−4.67 (−8.37,−2.66)	
MO	0.68 (0.44,0.98)	0.60 (0.45,0.86)	−0.09(−0.44,0.22)	0.78 (0.51,1.03)	0.59 (0.43,0.90)	−0.22 (−0.43,0.10)	
RBC (10^12^/L)	4.22 (3.95,4.50)	4.22 (3.94,4.46)	−0.01 (−0.26,0.20)	4.19 (4.01,4.42)	4.21 (4.00,4.41)	0.01 (−0.19,0.18)	
HB (g/L)	113.00 (106.00,120.00)	112.00 (105.00,119.25)	0.00 (−6.00,5.25)	112.00 (103.50,117.00)	111.00 (105.00,119.00)	−1.00 (−5.00,5.00)	
HCT	33.85 (31.50,35.60)	33.80 (31.80,35.90)	−0.05 (−2.40,1.60)	33.40 (31.75,35.40)	33.50 (31.80,36.00)	−0.10 (−1.63,2.13)	
PLT (10^9^/L)	342.50 (281.75,432.25)	493.00 (403.00,601.00)	147.00 (66.00,238.00)	352.00 (291.00,451.25)	530.00 (433.75,651.00)	162.50 (71.00,266.00)	
CRP (mg/L)	53.32 (22.48,91.90)	5.18 (2.12,9.30)	−51.96 (−81.15,−25.09)	56.80 (30.85,86.59)	4.58 (2.08,9.85)	−52.89 (−82.29,−28.79)	
ESR (mm/h)	59.88 ± 23.20	61.72 ± 20.91	−2.48 ± 22.10	63.75 ± 22.02	58.56 ± 23.21	−5.39 ± 22.06	
PCT (ng/ml)	0.35 (0.13,0.83)	0.10 (0.05,0.24)	−0.51 (−2.09,−0.07)	0.27 (0.14,0.81)	0.08 (0.05,0.22)	−0.63 (−2.12,−0.19)	
ALB (g/L)	37.65 ± 4.55	35.72 ± 4.01	−0.97 ± 5.06	37.98 ± 3.96	37.07 ± 3.62	−0.45 ± 4.16	
TBIL (μmol/L)	7.34 (5.60,10.18)	6.11 (4.10,8.00)	−3.50 (−8.90,−0.75)	6.48 (4.83,9.98)	4.80 (3.65,5.90)	−3.42 (−6.43,0.05)	
DBIL (μmol/L)	2.40 (1.60,3.75)	1.90 (0.93,3.68)	−2.50 (−4.90,−0.65)	2.10 (1.30,2.80)	1.40 (0.90,2.05)	−1.24 (−2.90,−0.55)	
IBIL (μmol/L)	4.50 (3.27,6.00)	3.50 (2.50,4.70)	−1.70 (−2.65,−0.42)	4.20 (3.00,5.40)	3.20 (2.58,3.86)	−1.61 (−3.15,0.25)	
ALT (U/L)	28.00 (18.00,66.00)	31.00 (21.00,52.25)	−29.00 (−99.25,1.25)	30.00 (14.50,50.00)	32.00 (19.50,52.50)	−22.00 (−99.00,1.00)	
AST (U/L)	28.00 (22.50,42.50)	37.00 (31.00,47.00)	5.00 (−27.00,13.00)	29.00 (21.50,40.00)	36.00 (30.00,50.00)	3.50 (−36.75,12.00)	
GGT (U/L)	21.00 (13.00,77.25)	44.00 (22.00,82.00)	−34.00 (−73.00,−4.50)	24.00 (13.00,59.00)	41.00 (26.25,92.00)	−25.50 (−77.00,−2.50)	
PT (s)^△^	13.70 ± 1.63	11.86 ± 1.28	−3.03 ± 2.01	13.35 ± 1.47	10.92 ± 1.21	−3.25 ± 1.71	^△^*P* = 0.039
PT%	81.00 (74.95,94.50)	102.00 (86.75,110.50)	24.20 (9.70,37.20)	88.50 (77.40,96.43)	112.40 (98.30,138.00)	26.50 (21.63,52.45)	
INR	1.09 (0.99,1.18)	0.96 (0.89,1.03)	−0.22 (−0.30,−0.10)	1.06 (1.00,1.15)	0.91 (0.84,0.96)	−0.24 (−0.30,−0.10)	
APTT (s)^△^	37.46 ± 7.52	30.26 ± 5.09	−12.53 ± 9.69	38.14 ± 9.66	25.80 ± 2.59	−20.83 ± 16.26	^△^*P* < 0.001
FIB (g/L)	5.85 ± 1.58	3.68 ± 1.05	−2.83 ± 1.22	5.96 ± 1.38	3.29 ± 1.03	−4.01 ± 1.73	
TT (s)	15.90 (15.15,16.70)	18.80 (17.60,19.50)	3.30 (2.40,5.20)	15.90 (15.48,16.85)	19.20 (17.70,19.80)	4.45 (3.30,4.70)	
DD (ng/ml)	1130 (780,1630)	830 (585,1560)	−610 (−1430,−290)	1180 (700,2190)	970 (520,3110)	−970 (−3230,−160)	
FDP (μg/mL)	4.05 (2.66,6.61)	2.62 (1.98,5.06)	−1.93 (−3.82,−0.46)	4.73 (3.08,7.88)	3.40 (2.40,4.82)	−2.04 (−7.14,−0.53)	

	IVIG therapy						*P* value
	Response			Nonresponse			
	Pre-IVIG	Post-IVIG	Delta	Pre-IVIG	Post-IVIG	Delta	
WBC (10^9^/L)	13.19 (10.29,16.17)	8.95 (7.62,11.53)	−3.51 (−6.76,−1.29)	13.19 (10.54,18.15)	8.75 (7.10,17.50)	−4.17 (−9.38,5.83)	
ANC (10^9^/L)	8.58 (5.80,10.88)	2.72 (1.68,4.22)	−5.55 (−7.98,−2.79)	10.87 (6.51,13.60)	2.92 (1.96,6.83)	−7.33 (−10.29,0.80)	
MO	0.70 (0.46,0.98)	0.60 (0.45,0.86)	−0.15 (−0.41,0.17)	0.92 (0.62,1.59)	0.51 (0.35,1.16)	−0.32 (−0.70,0.36)	
RBC (10^12^/L)^†^	4.23 ± 0.41	4.23 ± 0.38	0.01 ± 0.34	4.03 ± 0.47	3.84 ± 0.40	−0.15 ± 0.50	†*P* < 0.001
HB (g/L)^†^	113.00 (106.00,119.00)	112.00 (105.75,120.00)	0.00 (−5.00,6.00)	108.00 (98.50,116.00)	103.00 (93.00,111.50)	−5.00 (−6.00,1.00)	†*P* < 0.001
HCT^†^	33.71 ± 3.21	33.83 ± 3.10	0.12 ± 3.05	32.42 ± 2.66	31.83 ± 3.51	−0.59 ± 2.53	†*P* = 0.013
PLT (10^9^/L)	347.00 (282.00,432.50)	499.00 (409.50,623.00)	151.00 (68.00,239.50)	356.00 (259.00,505.00)	478.00(376.25,623.50)	119.00 (55.50,303.50)	
CRP (mg/L)	55.46 (25.36,87.69)	5.15 (2.34,9.50)	−52.74 (−81.23,−26.38)	47.28 (20.50,79.00)	5.50 (1.03,14.99)	−37.40 (−77.06,−17.19)	
ESR (mm/h)^†^	60.71 ± 22.61	59.86 ± 21.43	−3.62 ± 22.22	66.56 ± 27.40	73.60 ± 21.13	1.33 ± 19.87	†*P* = 0.017
PCT (ng/ml)	0.33 (0.14,0.99)	0.10 (0.05,0.24)	−0.63 (−2.22,−0.12)	0.39 (0.24,0.61)	0.07 (0.06,0.39)	−0.29 (−0.56,−0.13)	
ALB (g/L)	37.79 ± 4.30	36.13 ± 3.91	−0.65 ± 4.65	37.11 ± 5.42	35.86 ± 4.38	−2.33 ± 6.06	
TBIL (μmol/L)	7.16 (5.30,10.10)	5.31 (3.78,7.50)	−3.50 (−7.35,−0.75)	7.08 (5.65,11.38)	6.15 (4.30,10.35)	−1.65 (−8.13,−0.20)	
DBIL (μmol/L)	2.20 (1.55,3.55)	1.70 (0.90,3.20)	−2.00 (−3.40,−0.65)	2.60 (2.10,3.90)	1.80 (0.50,5.70)	−1.50 (−5.28,−0.50)	
IBIL (μmol/L)	4.40 (3.20,5.90)	3.20 (2.45,4.40)	−1.70 (−2.95,−0.22)	4.30 (3.70,8.00)	3.70 (3.30,5.05)	−1.45 (−2.85,0.78)	
ALT (U/L)	29.00 (17.00,59.00)	31.00 (21.00,52.25)	−23.00 (−99.50,1.50)	39.50 (15.50,63.75)	23.50 (17.50,55.00)	−9.50 (−45.25,−0.75)	
AST (U/L)	28.00 (22.00,41.00)	36.00 (31.00,46.75)	5.00 (−27.00,13.00)	26.50 (20.25,44.25)	42.00 (30.75,67.75)	−4.50 (−134.25,4.00)	
GGT (U/L)	22.50 (13.00,64.00)	44.00 (23.00,91.00)	−34.00 (−76.00,−4.50)	46.00 (11.00,117.75)	38.50 (23.50,95.50)	−7.50 (−57.50,12.00)	
PT (s)^†*^	13.65 (12.38,14.80)	11.50 (10.60,12.38)	−4.00 (−4.55,−1.80)	13.80 (12.05,15.00)	12.80 (11.85,14.05)	−1.00 (−0.2,−0.95)	†*P* = 0.040; ^*^P = 0.031
PT%^*^	83.55 (75.00,95.18)	103.00 (87.90,121.80)	27.40 (16.25,40.20)	77.00 (72.95,94.75)	99.25 (76.10,109.88)	22.25 (3.15,15.13)	
INR^*^	1.08 (0.99,1.18)	0.93 (0.88,0.99)	−0.27 (−0.30,−0.17)	1.07 (1.00,1.19)	1.00 (0.94,1.16)	−0.07 (−0.06,−0.03)	^*^P = 0.001
APTT (s)^†^	37.62 ± 8.11	28.50 ± 4.60	−15.57 ± 11.30	37.98 ± 8.90	35.53 ± 4.33	−3.30 ± 3.39	†*P* = 0.006
FIB (g/L)	5.97 ± 1.42	3.65 ± 0.92	−3.27 ± 1.20	4.94 ± 2.30	3.05 ± 1.84	−1.91 ± 2.02	
TT (s)	15.90 (15.18,16.70)	18.80 (17.70,19.60)	4.10 (2.50,4.95)	15.90 (15.75,17.05)	18.25 (16.98,22.60)	2.35 (1.23,5.55)	
DD (ng/ml)^※*^	1180 (790,1830)	830 (540,1560)	−610 (−1610,−290)	770 (560,820)	1640 (1140,2140)	1060 (510,1680)	^※^*P* = 0.031; ^*^P < 0.001
FDP (μg/mL)^※*^	4.37 (2.89,7.13)	2.62 (1.98,4.87)	−1.93 (−3.82,−0.61)	2.67 (1.93,3.12)	5.32 (3.92,6.73)	2.96 (0.72,5.03)	^※^*P* = 0.020; ^*^P < 0.001

	Coronary artery						*P* value
	Normal			CAAs			
	Pre-IVIG	Post-IVIG	Delta	Pre-IVIG	Post-IVIG	Delta	
WBC (10^9^/L)	13.21 (10.53,16.27)	8.97 (7.62,11.35)	−4.00 (−7.00,−1.26)	13.03 (9.38,15.59)	8.63 (6.68,13.09)	−2.67 (−3.89,−1.10)	
ANC (10^9^/L)	8.78 (5.86,11.19)	2.78 (1.72,4.25)	−5.71 (−8.08,−2.79)	7.33 (5.65,9.61)	2.64 (1.43,5.11)	−4.72 (−7.43,−1.87)	
MO	0.70 (0.46,0.98)	0.59 (0.44,0.85)	−0.12 (−0.43,0.21)	0.79 (0.49,1.27)	0.72 (0.46,1.04)	−0.24 (−0.55,0.09)	
RBC (10^12^/L)	4.21 ± 0.41	4.19 ± 0.39	−0.00 ± 0.35	4.30 ± 0.45	4.31 ± 0.40	0.02 ± 0.37	
HB (g/L)^#^	112.00 (105.00,119.00)	111.00 (105.00,119.00)	−1.00 (−6.00,5.00)	114.50 (107.00,126.00)	116.00 (108.50,125.00)	1.00 (−5.00,7.00)	^#^*P* = 0.009
HCT^#^	33.50 ± 3.10	33.57 ± 3.14	0.05 ± 2.98	34.95 ± 3.77	34.97 ± 2.96	0.30 ± 3.44	^#^*P* = 0.015
PLT (10^9^/L)	352.00 (282.00,440.50)	498.00 (413.25,621.50)	157.00 (68.00,238.50)	314.00 (284.00,376.00)	469.00 (383.00,649.50)	146.00 (65.50,257.00)	
CRP (mg/L)	55.39 (26.86,88.50)	4.80 (2.00,9.80)	−51.84 (−81.30,−26.77)	54.67 (11.38,81.49)	5.56 (2.78,8.63)	−49.83 (−78.50,−10.12)	
ESR (mm/h)	61.67 ± 22.92	61.17 ± 21.44	−3.56 ± 22.06	54.69 ± 21.97	56.82 ± 23.56	−0.94 ± 22.72	
PCT (ng/ml)	0.34 (0.14,0.89)	0.09 (0.05,0.23)	−0.58 (−1.96,−0.11)	0.25 (0.10,0.55)	0.15 (0.07,0.29)	−0.47 (−2.75,−0.16)	
ALB (g/L)	37.73 ± 4.40	35.91 ± 3.97	−1.04 ± 4.78	37.91 ± 4.09	37.14 ± 3.68	0.67 ± 4.76	
TBIL (μmol/L)	7.22 (5.23,10.00)	5.21 (3.83,7.50)	−3.60 (−8.90,−0.72)	7.03 (5.80,11.10)	6.70 (4.80,9.73)	−3.00 (−3.95,−0.30)	
DBIL (μmol/L)	2.25 (1.60,3.60)	1.70 (0.90,3.15)	−2.15 (−4.15,−0.78)	2.65 (1.50,2.90)	1.90 (0.90,4.00)	−1.05 (−2.03,−0.23)	
IBIL (μmol/L)	4.35 (3.15,5.90)	3.20 (2.48,4.25)	−1.75 (−3.13,−0.46)	4.45 (4.22,5.91)	3.74 (3.10,5.10)	−0.76 (−2.05,1.08)	
ALT (U/L)	29.00 (17.75,63.25)	31.00 (21.75,48.25)	−30.00 (−101.00,0.00)	25.50 (14.00,42.50)	31.00 (16.00,55.50)	−3.00 (−46.50,2.50)	
AST (U/L)	28.00 (22.00,42.00)	37.00 (31.00,47.00)	0.50 (−31.50,12.00)	26.50 (21.50,39.50)	36.00 (32.00,51.00)	9.00 (−5.50,21.00)	
GGT (U/L)	22.00 (13.00,72.50)	43.00 (23.50,86.50)	−33.00 (−76.00,−4.00)	32.00 (13.25,56.00)	53.00 (24.75,89.75)	−13.00 (−67.50,−3.50)	
PT (s)	13.65 (12.43,14.88)	11.70 (10.70,12.40)	−3.30 (−4.63,−1.03)	14.00 (11.10,14.70)	10.70 (10.10,12.40)	−3.44 (−4.30,−0.87)	
PT%	82.00 (74.93,93.70)	103.00 (86.80,121.20)	26.50 (11.68,36.50)	91.00 (78.00,97.70)	103.00 (100.15,128.70)	20.60 (−14.80,56.90)	
INR	1.08 (0.99,1.18)	0.95 (0.89,1.03)	−0.22 (−0.30,−0.11)	1.06 (0.92,1.17)	0.89 (0.85,0.98)	−0.22 (−0.30,0.03)	
APTT (s)	37.30 ± 7.48	29.24 ± 5.13	−13.86 ± 10.25	40.69 ± 12.61	28.38 ± 3.80	−16.50 ± 19.10	
FIB (g/L)	5.89 (4.91,6.91)	3.82 (3.06,4.32)	−3.00 (−3.56,−2.37)	6.56 (4.67,7.91)	2.86 (2.77,3.50)	−4.09 (−5.75,−2.38)	
TT (s)	15.90 (15.40,16.78)	18.60 (17.70,19.60)	3.60 (2.45,5.08)	15.90 (15.10,16.60)	19.00 (17.90,20.05)	4.07 (3.48,4.55)	
DD (ng/ml)^&^	1090 (770,1730)	850 (570,1820)	−530 (−1140,−250)	1340 (840,2880)	870 (480,1020)	−2900 (−3580,−2230)	^&^*P* < 0.001
FDP (μg/mL)	4.19 (2.84,6.82)	3.10 (2.04,5.11)	−1.85 (−3.08,−0.37)	4.75 (2.57,8.60)	2.50 (1.60,3.20)	−6.71 (−8.05,−5.37)	

^△^*P* < 0.05, significantly different between complete KD and incomplete KD after IVIG; ^※^*P* < 0.05, significantly different between IVIG-responsive and IVIG-nonresponsive group before IVIG; ^†^*P* < 0.05, significantly different between IVIG-responsive and IVIG-nonresponsive group after IVIG; *P < 0.05, significantly different in delta (δ) changes between IVIG-responsive and IVIG-nonresponsive group; #*P* < 0.05, significantly different between CAA group and non-CAA group after IVIG; ^&^*P* < 0.05, significantly different in delta changes between CAA group and non-CAA group after IVIG

### Coagulation profiles and IVIG therapy

Comparisons of laboratory characteristics between the IVIG-responsive and IVIG-nonresponsive group are shown in Table 2. Before IVIG therapy, serum DD and FDP were significantly higher in the IVIG-responsive group than those in the IVIG-nonresponsive group (*P* < 0.05). After IVIG therapy, RBC, HB and HCT were increased, whereas ESR, PT and APTT were decreased in the IVIG-responsive group significantly (*P* < 0.05). Overall, δPT and δINR were larger in the IVIG-responsive group, whereas δDD and δFDP were smaller in the IVIG-responsive group when compared with the IVIG-nonresponsive group (*P* < 0.05) (Fig. 2).

### Coagulation profiles and CAAs

Comparisons of laboratory characteristics between KD patients with and without CAAs are shown in Table 2. Before IVIG therapy, no significant differences were observed in all laboratory indicators regardless of whether CAAs existed or not (*P* > 0.05). After IVIG therapy, HB and HCT were significantly increased in the CAA group (*P* < 0.05). Overall, the CAA group showed higher δDD than the non-CAA group (*P* < 0.05) (Fig. 2). Moreover, δDD and δFDP were negatively correlated with the internal diameter of CA (DD and CA: *r* = −0.72, DD and LCA: *r* = −0.71; DD and RCA: *r* = −0.74, *P* < 0.05; FDP and CA: *r* = −0.54, FDP and LCA: *r* = −0.57; FDP and RCA: *r* = −0.53; *P* < 0.05).

## Discussion

Coagulation profiles can be helpful in identifying severe outcomes for patients with MIS-C. Although the pathogenesis is unclear, MIS-C has overlapping features with KD. In this circumstance, whether KD patients with impaired coagulation are likely to have severe outcomes should be noted by pediatric rheumatologists. The present study focuses on coagulation profiles in the acute episode of KD and summarizes three main findings. First, before therapy, coagulation disorders are more prone to appear in KD patients, and IVIG therapy facilitates to overcome them. Second, the larger δDD, δFDP and the smaller δPT, δINR predict IVIG nonresponsiveness. Third, the higher δDD and δFDP correlate with a higher risk for CAAs.

Accumulating evidence has demonstrated that coagulation disorders are highly prevalent in KD. According to the study by Chen et al*.* [15], the occurrence rate of hypercoagulation in KD was about 80%. The serum FIB was twofold higher (4.88 ± 0.71 g/L vs. 2.04 ± 0.48 g/L) and DD was three-fold higher (1.55 ± 1.74 mg/L vs. 0.48 ± 0.52 mg/L) in KD patients than in healthy children before IVIG therapy, respectively. Apart from the above indicators, our study still showed that higher PT, INR, APTT and FDP were noted in KD patients before IVIG therapy as compared with those after IVIG therapy and the healthy controls. The potential mechanisms of coagulation disorders in KD patients remain unclear and could be ascribed to hepatic dysfunction associated with inflammatory cell infiltration in sinusoids and portal areas, proliferation and/or swelling of Kupffer cells, fatty degeneration, and severe congestion [16, 17]. In the present study, 224 (71.57%) KD patients had varying degrees of hepatic dysfunction before therapy. Among them, hypoalbuminemia, elevated serum liver enzymes and hyperbilirubinemia were the most three common presentations, accounting for 72.8%, 38.7% and 6.4% of KD patients respectively. Liu et al. [18] found that TNF-α played a substantial role in promoting the expression of fibrinogen-like protein 2 which activated coagulation in CD31^+^ liver sinusoidal endothelial cell-like cells in vitro. Conversely, serum levels of DD in tnfα-/- mice were only 46% of that in tnfα^+^/^+^ mice.

High-dose IVIG plus aspirin is the first-line treatment for suppressing systemic inflammation in the acute episode of KD and potentially overcoming the coagulation disorders. In the present study, significant decreases were not only noted in WBC, ANC, CRP, ESR and PCT, but also in PT, INR, APTT, FIB, DD and FDP in KD patients after the initial therapy. Similarly, a retrospective study conducted by Sakurai et al*.* [19] indicated that PT and APTT had almost a onefold decrease, FDP and DD had almost a twofold decrease after therapy in KD patients. A retrospective study encompassing 210 KD patients by Mammadov et al*.* [20] demonstrated that FIB was subjected to almost a twofold decrease after therapy as compared with the time before therapy (3.93 ± 0.69 g/L vs. 7.59 ± 0.81 g/L). Our findings are partly in line with these above observations. As we all know, aspirin inhibits thrombin formation and thrombin-mediated coagulant reactions [21]. However, it remains unclear whether the correction of coagulation disorders is caused by aspirin or aspirin plus IVIG in KD. Given the overlapping clinical features of MIS-C and KD, IVIG is firstly recommended to most MIS-C patients [22]. A retrospective analysis conducted by Lee et al*.* [23] revealed that the high-dose IVIG (2 g/kg a dose) could reduce DD level in MIS-C patients by 50% average on day 7.2 after therapy. In this circumstance, we considered that the correction of coagulation disorders in KD may be due to the synergistic effects of IVIG and aspirin. In addition, it is possible that the significantly longer PT and APTT in patients with complete KD after IVIG therapy due to anticoagulant hesitancy occurs more often in their incomplete counterparts.

To date, several evaluation systems have been established to predict IVIG nonresponsiveness, which include many laboratory biomarkers such as some coagulation-related biomarkers. In a large prospective cohort study, Shao et al. [24] found that KD patients who have hypercoagulation during the acute phase could be at higher risk of developing IVIG nonresponsiveness, and cutoff values of coagulation profiles were as follows: PT ≥ 13.95 s, APTT ≥ 41.15s, DD ≥ 1480 ng/ml for IVIG nonresponsiveness before initial IVIG within 10 days from fever onset. Similarly, Kong et al. [25] demonstrated that a higher DD level had a sensitivity of 87.0% and a specificity of 56.3% for predicting IVIG nonresponsiveness at a cutoff point of 1.09 mg/l before IVIG therapy. However, the above studies only focused on the values of coagulation profiles before initial therapy. In the present study, we dynamically observed the changes of coagulation profiles and found that the median δPT and median δINR were smaller in the IVIG-nonresponders, whereas median δDD and median δFDP were larger in the IVIG-nonresponders when compared with IVIG responders. The underlying mechanisms regarding the association between coagulation indicators and IVIG nonresponsiveness in KD are not entirely clear. We consider that the larger fluctuations of DD and FDP reflect a more severe cytokine storm or even macrophage activation syndrome (MAS). MAS occurs rarely and is often associated with IVIG nonresponsiveness in KD. Latino et al. [26] noticed that 12 (1.9%) MAS were secondary to KD patients and all of them remained febrile for more than 48 h after the initial IVIG infusion; among them, elevated DD levels were present in 91.7% of KD-associated MAS patients. Therefore, δDD and δFDP are promising to be served as the predictors of IVIG nonresponsiveness, and our findings should be confirmed by a multicenter randomized clinical trials of larger samples in the future.

Besides association with IVIG nonresponsiveness, higher δDD also showed a significant correlation with CAAs. To date, the current evidence indicated that multi-factors are involved in mechanisms of CAAs. Among them, vascular endothelial cell damage associated with hyper-inflammation may lead to coagulative activation in the main CA [19, 27, 28]. In the present study, a significantly higher δDD was found in CAA group; and moreover, δDD and δFDP were negatively correlated with the internal diameter of CA. A previous case–control study encompassing 44 IVIG-nonresponders showed that DD had a sensitivity of 80.0% and a specificity of 78.3% at a cutoff value of 4800 ng/ml to predict acute phase CAAs in KD and all of these CAAs recovered by plasma exchange therapy [29]. As for those having received complete two doses of IVIG, although plasma exchange could decrease the acute risk of CAAs through the clearance of overexpressed DD, biologic agents are promising to serve as optimal option for refractory CAAs. A phase 3 randomized, double-blind, placebo-controlled trial [30] involving 196 KD patients demonstrated that the addition of infliximab (5 mg/kg) to standard therapy resulted in a twofold greater decrease in the left anterior descending CA Z scores compared with placebo at week 2 (SD −0.61 vs. −0.31). In another open label Phase II clinical study, Koné-Paut et al. [31] used anakinra to treat 16 KD patients who remained febrile 48 h after IVIG and discovered that the percentage of those who had a CA Z score > 2.5 decreased from 62.5% at the initial screening visit to 31% at day 45. However, these current studies rarely focus on the coagulation profiles, it remains unclear whether the biological agents have a improvement of refractory CAAs through rectifying coagulation disorders. Further studies are warranted to shed new light on this issue.

Limitations of our study include being a single center and retrospective in nature. Our hospital is a large tertiary medical center, which may lead to a selection bias due to a higher number of severely ill patients being admitted to this facility. Meanwhile, there may be unknown selection bias because this was a retrospective study.

## Data Availability

The datasets generated during and/or analyzed during the current study are available from the corresponding author on reasonable request.
